# What are the current outcomes of advanced gastrointestinal stromal tumors: who are the long-term survivors treated initially with imatinib?

**DOI:** 10.1007/s12032-013-0765-7

**Published:** 2013-11-12

**Authors:** Piotr Rutkowski, Jolanta Andrzejuk, Elżbieta Bylina, Czesław Osuch, Tomasz Świtaj, Anna Jerzak vel Dobosz, Urszula Grzesiakowska, Monika Jurkowska, Agnieszka Woźniak, Janusz Limon, Maria Dębiec-Rychter, Janusz A. Siedlecki

**Affiliations:** 1 Department of Soft Tissue/Bone Sarcoma and Melanoma, Maria Sklodowska-Curie Memorial Cancer Center and Institute of Oncology, Roentgena 5, 02-781 Warsaw, Poland; 2 Department of General Surgery, Jagiellonian University, Kopernika 40, 31-501 Cracow, Poland; 3 Department of Pathology, Maria Sklodowska-Curie Memorial Cancer Center and Institute of Oncology, Roentgena 5, 02-781 Warsaw, Poland; 4 Department of Radiology, Maria Sklodowska-Curie Memorial Cancer Center and Institute of Oncology, Roentgena 5, 02-781 Warsaw, Poland; 5 Department of Biochemistry and Molecular Biology, Institute of Rheumatology, Spartanska 1, 02-637 Warsaw, Poland; 6 Laboratory of Experimental Oncology, Catholic University of Leuven, Herestraat 49, Post 815, 3000 Louvain, Belgium; 7 Department of Biology and Genetics, Medical University of Gdansk, Debinki 1, 80-211 Gdańsk, Poland; 8 Center for Human Genetics, Catholic University of Leuven, Herestraat 49, Post 815, 3000 Louvain, Belgium; 9 Department of Molecular Biology, Maria Sklodowska-Curie Memorial Cancer Center and Institute of Oncology, Roentgena 5, 02-781 Warsaw, Poland

**Keywords:** Gastrointestinal stromal tumor, Imatinib, Prognosis, Predictive factors, Long-term survivors

## Abstract

The introduction of imatinib to clinical practice revolutionized therapy of advanced gastrointestinal stromal tumors (GIST), but its long-term results have been only just collected. We have attempted to identify factors related to the long-term survival. We have analyzed the data of 430 inoperable/metastatic/recurrent GIST patients treated with imatinib in reference centers, assessed the factors influencing the long-term overall survival (OS), and compared the outcomes in three periods of initiation of imatinib therapy during one decade (2001–2003, 2004–2006, 2007–2010). During analyzed time periods, we have found decrease in median largest tumor size at the start of imatinib therapy: 90.5 mm (2001–2003) versus 74 mm (2004–2006) versus 58 mm (2007–2010) (*p* = 0.002). Median progression-free survival (PFS) on 1st line imatinib was 37.5 months, without differences in PFS between three groups. Median OS was 5.8 years, 8-year OS rate was 43 %, and no difference in OS was demonstrated for patients treated in analyzed time periods. Independent good prognostic factors for longer OS were as follows: surgery of residual disease, initial WHO performance status 0/1, normal baseline albumin level, and the presence of exon 11 *KIT* mutations. Current median OS in advanced GIST reaches 6 years. The long-term survivors were characterized by smaller maximal tumors at imatinib start, better blood tests results, better performance status, and the surgical removal of residual disease. The latter might reduce the impact of tumor size and equalize the long-term results of therapy during last decade from introduction of imatinib. After introduction of subsequent lines of therapy (as sunitinib), the effect of primary mutational status on the long-term OS is also less visible.

## Introduction

The introduction of imatinib to therapy of advanced gastrointestinal stromal tumors (GIST) has dramatically improved the outcomes of these tumors [1, 2]. Imatinib as tyrosine kinase inhibitor inhibiting KIT/PDGFRA (platelet-derived growth factor receptor alpha) and their downstream signaling cascade in GIST cells is currently standard of care in the first-line therapy of inoperable and/or metastatic tumors [3], and became the model of targeted therapy of solid tumors. Its efficacy has been also proven recently in adjuvant setting after resection of primary high-risk tumors [4, 5]. However, a majority of patients eventually develop clinical resistance to imatinib. Over the last few years, major progress has been made in elucidating the mechanism of disease progression and resistance to imatinib such as secondary mutations in KIT and/or PDGFRA kinase domains. Currently, the sole-approved second-line drug is sunitinib—a multi-targeted agent [6]. Moreover, a number of new generation tyrosine kinase inhibitors (as regorafenib, registered recently in USA), alone or in combination, are being evaluated at present alongside treatment options alternative to inhibiting the KIT signaling pathway [7].

There are limited data regarding the long-term outcomes of metastatic GIST outside the clinical trials in routine practice. The aim of this large contemporary series of inoperable/metastatic GIST was to identify factors related to progression-free and overall survival (OS) of patients starting imatinib therapy as well as to attempt to identify the factors related to subgroup of patients with the long-term survival.

## Patients and methods

### Patients

In this observational study, we analyzed collected prospectively data of 430 consecutive patients treated initially with imatinib mesylate (according to approved registration) due to inoperable and/or metastatic histologically confirmed, CD117-positive GIST, who were treated or referred to tertiary sarcoma center within framework of the Polish Clinical GIST Registry between September 1, 2001 and December 31, 2010. Each patient provided informed consent for the study. The study has been approved by the local Bio-Ethics Committee according to Good Clinical Practice Guidelines. Patients did not undergo any further selection. The distribution of clinical and pathological data of patients included in the study is listed in Table 1. There were 226 male and 204 female patients, with median age at the start of imatinib therapy 58 years (range 15–89).

**Table 1:** Characteristics of 430 patients treated initially with imatinib due to advanced GIST

Clinicopathological features	No. of patients
Total number of patients	430 (100 %)
Age (years) at the start of imatinib therapy	
Median (range) mean	58 (15–89) 57
≤40	42 (9.8 %)
>40	388 (90.2 %)
Gender	
Female	204 (47.4 %)
Male	226 (52.6 %)
The period of initiation of imatinib therapy (years)	
2001–2003	100 (23.3 %)
2004–2006	166 (38.6 %)
2007–2010	164 (38.1 %)
Primary tumor site	
Stomach	151 (35.1 %)
Duodenum	23 (5.4 %)
Small bowel	179 (41.6 %)
Large bowel/rectum	34 (7.9 %)
Other or intraperitoneally with unknown primary origin	43 (10.0 %)
The maximal diameter of the largest tumor (mm)	
Median (range)	73 (10–400)
≤50	108 (25.1 %)
>50–100	105 (24.4 %)
>100	118 (27.4 %)
Data not available	99 (23.0 %)
Resection of residual disease during imatinib therapy	
Yes	94 (21.9 %)
No	336 (78.9 %)
Presence of liver metastases at imatinib start	
Yes	220 (51 %)
No	210 (49 %)
Tumor genotype^a^	
*KIT* exon 11	139 (63.2 %)
*KIT* exon 9	29 (13.1 %)
Wild type	34 (15.5 %)
*PDGFRA* exon 18 D842V	9 (4.1 %)
Other	9 (4.1 %)
Baseline albumin level	
Low (<35 g/l)	58 (13.5 %)
Normal (>35 g/l)	230 (53.5 %)
Data not available	142 (33.0 %)
Baseline hemoglobin level	
Low (<11 g/100 ml)	65 (15.1 %)
Normal (≥11 g/100 ml)	278 (64.7 %)
Data not available	87 (20.2 %)
Baseline neutrophils count	
High (>5 × 10^9^/l)	72 (16.7 %)
Normal (<5 × 10^9^/l)	262 (60.9 %)
Data not available	96 (22.3 %)
Performance status (WHO score)	
Poor ≥2	75 (17.5 %)
Good <2	272 (63.2 %)
Data not available	83 (19.3 %)

^a^Mutational status was evaluated in 220 cases

All but eight patients (who started imatinib therapy from 800 mg/day) were treated with imatinib in initial dose of 400 mg daily. All patients were followed carefully with median follow-up time for survivors of 51 months. The objective response of GIST to imatinib therapy was evaluated with serial CT examinations (performed every 2–3 months), according to Response Evaluation Criteria in Solid Tumors (RECIST) version 1.0 [8]. In doubtful cases of progressive disease, additional Choi’s criteria were applied [9]. In the case of progression or unacceptable toxicity (three cases), patients were treated with imatinib at the higher doses (600–800 mg daily) or the therapy was immediately changed to sunitinib. One hundred and eighty-eight progressing patients were thereafter treated with sunitinib (since 2005). Subsequently, patients were treated according to decision of treating physician with either best supportive care, experimental therapy (nilotinib or regorafenib), off-label use of sorafenib, reintroduction of imatinib, or chemotherapy.

Multidisciplinary team evaluated possibility of surgical treatment of residual lesions (liver and/or intraperitoneal metastases), which had been estimated as resectable after maximal response to imatinib (as described previously) [10].

Genomic screening was performed for the presence of the *KIT* (exons 9, 11, 13, and 17) or *PDGFRA* (exons 12, 14, and 18) genes mutation in randomly selected 220 cases, based on DNA isolated from paraffin-embedded or fresh-frozen imatinib-naive tumor tissues, as previously described [11].

### Statistical analyses

All statistical analyses were performed using R 2.10.1 statistical program.^1^ For the survival analysis, the Kaplan–Meier estimator was used with the log-rank tests for bivariate comparisons. The primary objective of the study was to assess the OS of advanced GIST treated initially with imatinib as well as to identify the factors related to longer OS time. The secondary objectives were to estimate progression-free survival (PFS) on imatinib therapy and to describe the factors related to improved PFS time. OS time was calculated from the date of the start of imatinib treatment to the date of the most recent follow-up or death. PFS time was calculated from the date of the start of imatinib treatment to the date of the most recent follow-up, or progression or death due to the disease. The survival was assessed with respect to the following variables: demographic data (age at the start of imatinib therapy ≤40 or >40 years; gender), the period of initiation of imatinib therapy (2001–2003 vs. 2004–206 vs. 2007–2010), primary tumor genotype (*KIT* exon 11, *KIT* exon 9, *PDGFRA* exon 18 D842V mutations, wild type, and other cases), the maximal diameter of the largest tumor at imatinib start, the presence versus absence of liver metastases, primary tumor site (gastric vs. duodenum vs. small bowel—ileum or jejunum vs. large bowel vs. other or intraperitoneally with unknown primary origin), baseline (1–7 days before start of imatinib therapy) albumin level (low <35 g/l vs. normal >35 g/l), baseline (1–7 days before start of imatinib therapy) hemoglobin level (low <11 g/100 ml or normal ≥11 g/100 ml), baseline (1–7 days before start of imatinib therapy) neutrophils count (high >5 × 10^9^/l vs. normal <5 × 10^9^/l), baseline (1–7 days before start of imatinib therapy) performance status according to World Health Organization (WHO) (good: 0–1 vs. poor ≥2), and the fact of resection of GIST residual disease during imatinib therapy. In multivariate analysis of the factors associated with PFS, we used Cox proportional hazards models, applying the stepwise model building procedure that included all covariates significant at 20 % level in bivariate analysis. The best model was based on Akaike’s criterion. The differences were considered statistically significant if the *p* values were <0.05.

## Results

### Clinicopathological and mutational data

During analyzed time periods, we have found decrease in median largest tumor size at the start of imatinib therapy: 90.5 mm (2001–2003) versus 74 mm (2004–2006) versus 58 mm (2007–2010) (*p* = 0.002).

The distribution of patients according to the tumor mutational status is shown in Table 1. In total, 85 % of cases revealed a *KIT* or *PDGFRA* mutation (63 %—exon 11 *KIT*, 13 %—exon 9 *KIT*, 4.1 %—exon 18 *PDGFRA* D842V, and 4.1 %—other types of mutation).

### Progression-free survival on imatinib therapy

Progression of disease during imatinib therapy was observed in 246 cases (57 %). Median PFS was 37.5 months, and estimated 5- and 8-year PFS rates were 37.0 and 27 %, respectively.

**Fig. 1:**
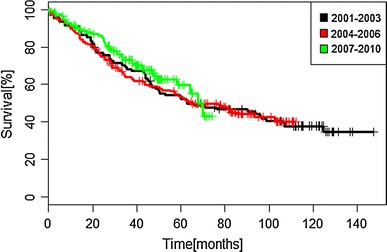
Progression-free survival according to periods of time of initiation of imatinib therapy

We have not observed significant differences in PFS between three analyzed periods of time (Fig. 1).

In univariate analysis, the following factors correlated with shorter PFS (Table 2a): lack of resection of residual disease during imatinib therapy, primary tumor located in duodenum or intraperitoneally with unknown primary origin, the maximal tumor diameter of the largest tumor >100 mm, tumor mutation other than *KIT* exon 11, the younger age, low baseline albumin level, high baseline neutrophils count, low baseline hemoglobin level, and poor performance status.

**Table 2:** Univariate analysis for PFS (a) and OS (b) in the entire cohort of patients

Factor	Subgroup	PFS (months)		5-year PFS	95 % CI	8-year PFS	95 % CI	*p* value
		Median	95 % CI					
(a)								
Gender	Female	43.6	33.0–49.4	37.7	30.7–46.4	30.6	23.6–39.7	0.508
	Male	34.8	28.4–45.2	36.2	29.6–44.2	24.1	17.7–32.6	
Age	≤40	23.7	15.4–66.8	32.9	20.4–53.1	20.3	9.6–42.8	0.246
	>40	38.8	32.3–47.3	37.4	32.1–43.4	28.0	22.8–34.4	
The period of initiation of imatinib therapy (years)	2001–2003	45.3	36.4–66.7	42.3	33.3–53.7	32.5	24.1–44.0	0.273
	2004–2006	39.6	30.8–50.2	35.8	28.5–44.8	24.5	17.9–33.6	
	2007–2010	30.0	26.3–42.3	38.1	30.0–48.5	38.1	30.0–48.5	
Resection of residual disease during imatinib therapy	Yes	NA	64.9–NA	62.2	52.1–74.4	50.4	39.4–64.6	<0.001
	No	27.5	24.2–34.1	29.0	23.8–35.4	19.7	14.8–26.3	
Primary tumor site	Stomach	35.5	7.4–38.5	42.1	33.9–52.3	31.8	23.5–43.0	
	Duodenum	21.2	8.13–NA	12.6	2.3–69.1	12.6	2.3–69.1	0.0002
	Small bowel	40.9	33.6–48.1	35.8	28.7–44.7	25.4	18.4–35.0	
	Large bowel/rectum	66.6	45.2–NA	59.1	42.4–82.3	47.3	30.0–74.4	
	Other or intraperitoneally with unknown primary origin	15.9	7.4–38.5	15.8	6.6–38.1	11.9	4.2–33.8	
Presence of liver metastases at imatinib start	Yes	40.7	33.2–50.9	37.1	30.5–45.1	23.8	17.6–32.2	0.858
	No	33.0	27.8–45.4	36.7	29.8–45.3	31.3	24.3–40.3	
The maximal diameter of the largest tumor (mm)	≤50	56.5	44.9–86.4	49.1	38.4–62.6	31.9	21.2–48.0	0.003
	>50–100	66.6	43.6–NA	53.2	43.0–65.8	39.0	28.5–53.6	
	>100	27.3	21.8–40.9	27.8	19.8–39.2	21.8	14.3–33.1	
Tumor genotype	Wild type	20.6	11.5–NA	34.0	19.2–60.2	27.2	13.2–55.9	0.005
	Exon 11 *KIT*	47.3	36.4–66.6	41.9	33.5–52.5	31.6	23.1–43.2	
	Exon 9 *KIT*	20.9	14.0–38.8	18.6	7.9–43.7	18.6	7.9–43.7	
	Exon 18 *PDGFRA* D842V	2.3	1.7–NA	22.2	6.5–75.4	22.2	6.5–75.4	
	Other	NA	5.2–NA	57.1	30.1–100.0	57.1	30.1–100.0	
Baseline albumin level	Normal	59.9	44.2–80.4	49.3	42.7–57.0	36.4	29.5–44.9	<0.001
	Low	23.7	19.2–34.8	15.4	7.8–30.1	12.8	6.0–27.5	
Baseline neutrophils count	Normal	56.5	45.3–80.4	49.2	42.4–57.1	36.8	29.8–45.5	<0.001
	High	20.2	16.9–28.7	20.9	12.5–34.7	15.6	8.2–29.9	
Baseline hemoglobin level	Normal	56.5	44.9–75.7	48.7	42.0–56.4	36.0	29.1–44.4	<0.001
	Low	19.4	15.9–26.6	18.0	9.9–32.7	15.0	7.4–30.1	
Performance status (WHO score)	Good <2	59.9	47.3–75.7	49.4	43.0–56.9	37.0	30.4–45.2	<0.001
	Poor ≥2	16.3	11.5–20.4	9.2	3.9–21.6	6.9	2.5–19.2	
All		37.5	31.7–45.2	37.0	32.1–42.7	27.2	22.3–33.2	–
(b)								
Gender	Female	78.3	60.6–NA	57.2	50.0–65.5	47.3	39.5–56.7	0.706
	Male	64.3	51.5–95.7	52.9	45.9–60.9	39.8	32.0–49.4	
Age	≤40	NA	55.2–NA	63.4	49.2–81.8	57.6	42.0–79.0	0.152
	>40	66.1	58.8–93.8	54.0	48.5–60.0	41.6	35.6–48.6	
The period of initiation of imatinib therapy (years)	2001–2003	62.9	46.8–107	52.1	42.9–63.3	42.9	33.9–54.4	0.589
	2004–2006	64.9	51.5–NA	53.4	45.9–62.1	42.5	34.7–52.1	
	2007–2010	70.0	58.1–NA	59.6	49.7–71.6	43.2	28.2–66.0	
Resection of residual disease during imatinib therapy	Yes	NA	NA–NA	80.3	71.8–89.9	67.4	56.6–80.3	<0.001
	No	55.2	43.9–70	46.8	41.0–53.4	35.6	29.3–43.2	
Primary tumor site	Stomach	93.4	45.1–NA	53.6	45.3–63.4	45.8	36.2–57.8	
	Duodenum	61.8	55.2–NA	57.1	35.9–91.1	30.5	10.9–84.9	0.002
	Small bowel	70.8	60.6–102.4	58.2	50.6–67.0	41.0	32.7–51.4	
	Large bowel/rectum	NA	46.6–NA	64.9	49.1–85.6	64.9	49.1–85.6	
	Other or intraperitoneally with unknown primary origin	21.9	19.0–62.9	29.1	16.4–51.7	25.5	13.6–47.9	
Presence of liver metastases at imatinib start	Yes	90.1	60.6–NA	57.0	50.1–64.9	44.9	37.1–54.4	0.249
	No	64.9	50.2–98.5	52.7	45.3–61.2	42.0	34.4–51.4	
The maximal diameter of the largest tumor (mm)	≤50	NA	98.5–NA	78.3	69.1–88.7	63.5	51.2–78.8	<0.001
	>50–100	124.7	96.7–NA	65.3	55.5–76.7	61.0	50.6–73.6	
	>100	46.8	37.9–63.7	39.6	30.4–51.6	27.2	18.2–40.6	
Tumor genotype	Wild type	66.1	14.23–NA	52.9	37.4–74.7	47.0	31.0–71.2	0.157
	Exon 11 *KIT*	82.4	60.60–NA	59.6	51.0–69.7	47.0	37.5–58.9	
	Exon 9 *KIT*	78.3	55.20–NA	60.2	43.5–83.5	42.6	25.5–71.1	
	Exon 18 *PDGFRA* D842V	15.5	8.33–NA	33.3	13.2–84.0	33.3	13.2–84.0	
	Other	NA	17.20–NA	66.7	37.9–100.0	66.7	37.9–100.0	
Baseline albumin level	Normal	124.7	96.7–NA	68.7	62.5–75.5	58.1	50.7–66.6	<0.001
	Low	37.8	31.3–50.2	25.4	15.6–41.3	15.2	7.6–30.8	
Baseline neutrophils count	Normal	124.7	95.7–NA	67.6	61.2–74.7	56.7	49.1–65.4	<0.001
	High	37.8	29.4–49.3	31.9	21.8–46.6	22.8	13.8–37.7	
Baseline hemoglobin level	Normal	106.4	93.4–NA	65.8	59.5–72.7	54.1	46.8–62.6	<0.001
	Low	38.8	31.7–52.8	29.9	19.2–46.6	24.5	14.5–41.3	
Performance status (WHO score)	Good <2	124.7	96.7–NA	70.0	64.2–76.5	58.4	51.3–66.5	<0.001
	Poor ≥2	27.5	21.9–43.4	12.7	6.2–26.3	8.5	3.4–21.3	
All		70.8	59.9–95.7	54.9	49.8–60.6	43.4	37.7–50.0	–

*CI* confidence interval, *NA* not applicable

In the multivariate analysis (final Cox model), we identified the following independent predictive factors, which correlated with poorer PFS (Table 3a): worse baseline WHO performance status, high baseline neutrocyte count, low baseline hemoglobin level, younger age, the lack of resection of residual disease, primary tumor site, and tumor mutation other than *KIT* exon 11.

**Table 3:** Multivariate analysis of prognostic factors for PFS (a) and OS (b)

Factor	Subgroup	HR	95 % CI	*p* value
(a)				
Age	>40	1.00		
	≤40	1.802	1.1875–2.7347	0.005
Resection of residual disease during imatinib therapy	Yes	1.00		
	No	0.3539	0.2420–0.5175	<0.001
Primary tumor site	Duodenum	1.00		
	Small bowel	0.7208	0.4097–1.2682	0.256
	Large bowel/rectum	0.3959	0.1793–0.8741	0.022
	Stomach	0.4711	0.2576–0.8613	0.014
	Other or intraperitoneally with unknown primary origin	1.315	0.6809–2.5401	0.415
Tumor genotype	Wild type	1.00		
	Exon 11 *KIT*	0.5897	0.3458–1.0055	0.052
	Exon 9 *KIT*	1.2	0.6183–2.3292	0.589
	Exon 18 *PDGFRA* D842V	4.102	1.6520–10.1851	0.002
	Other	0.9746	0.2781–3.4154	0.968
Baseline neutrophils count	Normal	1.00		
	High	1.72	1.1724–2.5248	0.006
Baseline hemoglobin level	Normal	1.00		
	Low	1.592	1.0396–2.4385	0.032
Performance status (WHO score)	Good <2	1.00		
	Poor ≥2	2.79	1.8647–4.1755	<0.001
(b)				
Resection of residual disease during imatinib therapy	Yes	1.00		
	No	0.3179	0.20001–0.5052	<0.001
Primary tumor site	Duodenum	1.00		
	Small bowel	1.2	0.59257–2.4317	0.612
	Large bowel/rectum	0.9214	0.36381–2.3337	0.863
	Stomach	0.8693	0.41325–1.8285	0.712
	Other or intraperitoneally with unknown primary origin	2.437	1.08447–5.4745	0.031
Presence of liver metastases at imatinib start	Yes	1.00		
	No	1.387	1.01151–1.9029	0.042
The maximal diameter of the largest tumor (mm)	≤50	1.00		
	>50–100	1.04	0.60162–1.7980	0.888
	>100	1.636	0.97002–2.7583	0.065
Tumor genotype	Wild type	1.00		
	Exon 11 *KIT*	0.4466	0.24163–0.8254	0.01
	Exon 9 *KIT*	0.6156	0.28451–1.3318	0.218
	Exon 18 *PDGFRA* D842V	3.049	1.142–8.1414	0.026
	Other	0.4076	0.09017–1.8429	0.244
Baseline albumin level	Normal	1.00		
	Low	2.415	1.48174–3.9363	0.0004
Baseline hemoglobin level	Normal	1.00		
	Low	1.007	0.60318–1.6801	0.979
Performance status (WHO score)	Good <2	1.00		
	Poor ≥2	2.427	1.53092–3.8491	0.0002

*HR* hazard ratio, *CI* confidence interval

### Overall survival

**Fig. 2:**
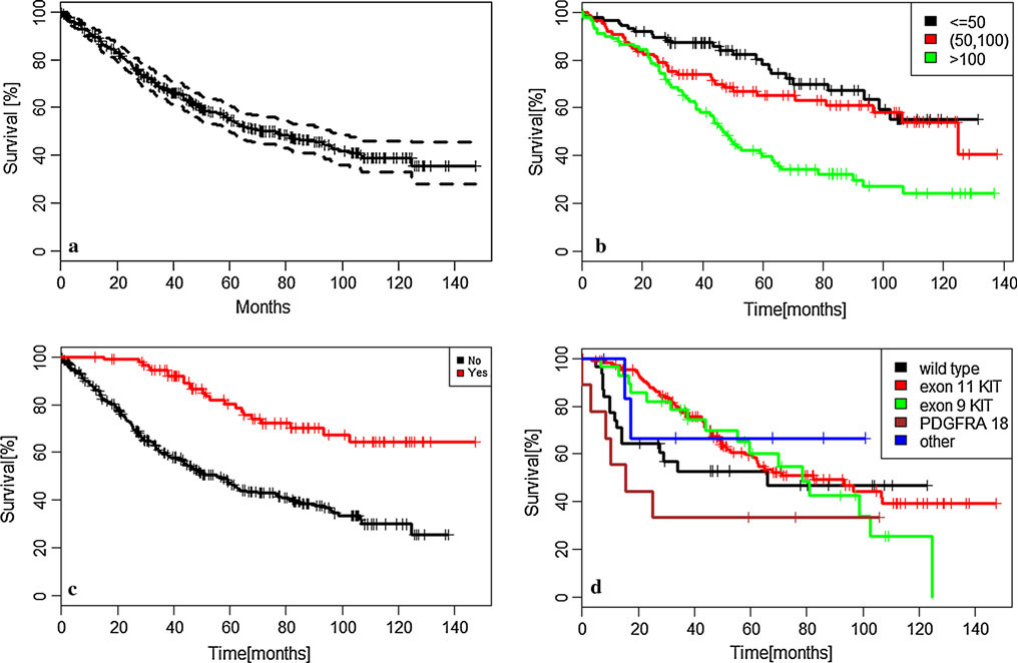
Overall survival: in the entire group of patients (**a**); according to maximal diameter of tumor at start of imatinib therapy in mm (**b**); according to the lack of resection of residual disease during imatinib therapy (**c**); and according to initial mutational status (**d**)

At the time of analysis, 241 (56 %) patients were alive. Median OS was 37.5 months, and estimated 5- and 8-year PFS rates were 57 and 47 %, respectively (Fig. 2a).

We have not observed significant differences in OS between three analyzed periods of time (data not shown).

The following factors significantly influenced OS in bivariate analysis (Table 2b): poor baseline WHO performance status ≥2, baseline high neutrocyte count, baseline low albumin level, low baseline hemoglobin level, the maximal diameter of the largest tumor >10 cm (Fig. 2b), and the lack of resection of residual disease during imatinib therapy (Fig. 2c). Patients with primary tumors carrying mutation D842V in exon 18 *PDGFRA* had substantially shorter OS reaching median OS only 15.5 months (Fig. 2d).

The following factors were found to be independent predictors of better OS according to multivariate analysis (Table 3b): good baseline WHO performance status, normal baseline albumin level, the resection of residual disease during imatinib therapy, the presence of exon 11 *KIT* mutations, and (with borderline significance) the maximal tumor diameter of the largest tumor >10 cm.

## Discussion

Our data comprise the largest series of advanced GIST patients treated in routine practice and were collected prospectively in tumor-type-specific national registry with the long follow-up. Several conducted clinical trials confirmed high efficacy of imatinib in the treatment of inoperable/metastatic GIST [1, 2, 12, 13] as compared to historical clinical data with median survival of patients being 10–19 months [3, 14], with the current survival being strikingly superior [15]. The median OS reported until now in few studies reached only from 4.0 to 6.4 years [12, 13, 16–21]. Our data confirm this superior survival. Moreover, although the spectacular response to imatinib therapy is time-limited and followed by the development of secondary resistance (after initial stabilization or response) in the majority of patients, still 1/4 of patients have not been progressing at 8 years of therapy with imatinib. The current PFS in our series on first-line therapy with imatinib is approximately 3 years, what is almost the same as in the recent Taiwanese one-institution study [20], and it did not improve significantly over decade from the turning point of introduction of imatinib to clinical practice. However, we have found systematically decrease in maximal tumor burden during this period of time, what is probably related to better follow-up of patients after resection of primary tumor and earlier detection of recurrent disease. The cut-off value for tumor bulk which had significantly inferior impact on PFS and OS was 10 cm in our series defined as the single largest size of measurable lesions. It confirms the previous data that largest tumor may be related to higher likelihood of development of resistant clones and secondary mutations [13, 20, 22–25], and it underlines the utility of tumor bulk assessment by the single largest lesion.

We have previously identified some predictive factors for the benefit of imatinib therapy in terms of inhibition of disease progression in advanced GIST [26]. Also, van Glabbeke and co-authors [27] had reported data on distinctive predictive clinicopathological factors for initial and late resistance to imatinib in advanced GISTs, but this analysis did not include the genotyping of the tumor as well as the strategy of removal of residual disease during therapy with tyrosine kinase inhibitors. Currently, we have expanded the variables predictive for the long-term outcomes and survival of inoperable and/or metastatic GISTs treated initially with imatinib. Based on these results of univariate and multivariate analyses, we can identify the patients’ factors which are related to benefits of longer survival: initial better performance status and laboratory test results (especially normal albumin level), primary tumor genotype (exon 11 *KIT* mutants and genotype other than exon 18 *PDGFRA* D842V), the smaller maximal size of the largest tumor, and resection of residual disease during imatinib therapy. These factors may account for the basis for development of the nomogram for PFS and OS [28]. Laboratory factors as high granulocyte count, low hemoglobin level, or low albumin level together with poor general performance status were previously implied as predictive factors for resistance to imatinib therapy [12, 13, 17, 18, 23, 26, 27]. Consistently with the results of present series, these factors can be related to generally more advanced and aggressive tumors, with higher inflammatory component influencing pharmacokinetics of the drug [13, 17, 27, 29, 30].

In patients with available data on tumor genotype, we found consistently with results already reported [12, 31–34] that the mutational status had significant impact on prognosis, with the best results for *KIT* exon 11 mutants in terms of PFS and OS. For OS, the effect of presence of *KIT* exon 11 as compared to *KIT* exon 9 mutations was less evident, which may be related to the impact of subsequent lines of therapy (mainly with sunitinib, which is more active for *KIT* exon 9 mutants [35]. Notably, according to Blanke et al. [13], the effect of exon 11 *KIT* mutations on OS mainly resulted from their strong effect during the first 30 months of treatment. We could not analyze the influence of higher dose of imatinib on PFS in subgroup of patients with *KIT* exon 9 mutations because all but eight patients started therapy from registered dose of 400 mg. The available data (from EORTC-ISG-AGITG 62005 trial and meta-analysis with S0033) have shown that the response of patients with exon 9 *KIT* mutations depends on the dose of the drug and that these patients under higher does (800 mg daily) of imatinib demonstrate significant improvement of PFS as compared to a standard dose of 400 mg daily (without impact on OS) [17, 36]. Furthermore, although the presence of *PDGFRA* D842V mutation is related to more indolent disease in primary resectable GIST [37], it is poor prognostic factor in advanced disease, as this mutation is insensitive to commonly used tyrosine kinase inhibitors (including imatinib and sunitinib) [33, 38] and is responsible for primary resistance to imatinib.

Surgery of residual disease in situation of absence of disease progression was found as the most independent prognostic factor for better outcomes in advanced GIST. Some studies have already reported favorable outcomes of surgery in responding patients [10, 39–42]. The present series demonstrate clear improvement in the long-term term survival in the group of patients operated after response to imatinib therapy (median PFS and OS were not reached). Although we cannot exclude selection bias as the role of surgery in metastatic GIST has never been confirmed in prospective study (as the initiated studies failed because of slow recruitment) [43], we still believe in real impact of this strategy on natural course of the disease. It can theoretically prolong durable remission, because the excision of the tumor is performed before the development of imatinib resistance, and thus, the risk of resistant clone selection is reduced. We have rather liberally used surgical removal of residual disease after individual decision made on multidisciplinary tumor board, as more than 20 % patients responding to systemic therapy underwent surgery following imatinib therapy, what might reduce the impact of initial tumor size at advanced setting.

Although imatinib is the most important therapy in GIST, predominantly influencing survival in advanced disease, the difference between median PFS and OS on initial therapy with imatinib in our study is more than 2.5 years. There are several reasons for these results, suggesting relative efficacy of salvage therapy after imatinib failure. Multidisciplinary approach after progression on initial dose of imatinib includes increase of the dose of imatinib to 800 mg daily [44], surgical resection, or ablation of focally progressive disease [10, 39, 42], using therapy with alternative receptor tyrosine kinase inhibitors (as second-line registered multi-targeted tyrosine inhibitor sunitinib, or further line therapy with sorafenib or regorafenib, which recently has been approved in USA) [7, 45]. We have recently analyzed the results of sunitinib therapy in series of 137 patients after failure of imatinib therapy and demonstrated survival exceeding 1.5 years from start of sunitinib [36], as well as we have also proven that contrary to imatinib, tumors initially (pre-imatinib treatment) bearing *KIT* exon 9 mutation or with wild-type genotype have a higher chance to respond to sunitinib. We also actively used therapy with alternative tyrosine kinase inhibitors after progression on imatinib and sunitinib (not only best supportive care), which also may be related to better OS observed in our study [46] and lack of differences between different periods of treatment.

To summarize, the current median survival in advanced GIST reaches 6 years. The long-term survivors (with OS exceeding 5 years) were characterized by smaller maximal tumors at start of imatinib therapy, better laboratory tests results, better performance status, and more commonly use of surgical removal of residual disease. The latter might reduce the impact of tumor size and equalize the long-term results of therapy during last decade from the introduction of imatinib. In addition, after introduction of subsequent lines of therapy, the effect of primary mutational status (with exception of *PDGFRA*-D842V) on long-term OS is less visible.

## Footnotes

1 R Development Core Team; http://www.R-project.org.
